# Four-Dimensional-Printed Microrobots and Their Applications: A Review

**DOI:** 10.3390/mi14081607

**Published:** 2023-08-15

**Authors:** Bobby Aditya Darmawan, Jong-Oh Park, Gwangjun Go, Eunpyo Choi

**Affiliations:** 1 Korea Institute of Medical Microrobotics, 43-26, Cheomdangwagi-ro 208-beon-gil, Buk-gu, Gwangju 61011, Republic of Korea; bobbyadit93@kimiro.re.kr (B.A.D.); jop@kimiro.re.kr (J.-O.P.); 2 Department of Mechanical Engineering, Chosun University, 309 Pilmun-daero, Dong-gu, Gwangju 61452, Republic of Korea; 3 School of Mechanical Engineering, Chonnam National University, 77 Yongbong-ro, Buk-gu, Gwangju 61186, Republic of Korea

**Keywords:** 4D-printed, microrobots, stimuli-responsive, shape reversible, medical, nonmedical, applications

## Abstract

Owing to their small size, microrobots have many potential applications. In addition, four-dimensional (4D) printing facilitates reversible shape transformation over time or upon the application of stimuli. By combining the concept of microrobots and 4D printing, it may be possible to realize more sophisticated next-generation microrobot designs that can be actuated by applying various stimuli, and also demonstrates profound implications for various applications, including drug delivery, cells delivery, soft robotics, object release and others. Herein, recent advances in 4D-printed microrobots are reviewed, including strategies for facilitating shape transformations, diverse types of external stimuli, and medical and nonmedical applications of microrobots. Finally, to conclude the paper, the challenges and the prospects of 4D-printed microrobots are highlighted.

## 1. Introduction

Microrobots can be defined as tiny (nano–microscale) self-propelled or externally propelled artificial devices that are designed to execute special tasks [1,2]. Similar to how conventional human-scale robotics has revolutionized every contemporary industry, the development of microrobotic systems that can execute useful tasks can potentially advance diverse fields [3]. Over the past few decades, many scientists have researched microrobots extensively, including the associated design strategies, fabrication techniques, propulsion mechanisms, motion behaviors, and demonstrative applications [4]. However, despite substantial advances in medical technology, most sampling, surgery, and treatment procedures remain invasive, and they may have several side effects [5]. In this context, there is a need to develop microrobots that can reach small, targeted areas wirelessly. The ability to handle small objects within extremely small volumes of fluid and to manipulate, move, and reconfigure components by using microrobots is one of the advantages of this approach [6]. In addition, it takes more than simply miniaturizing existing robotic technologies to build and operate microscopic robots, which must be modeled and designed based on microorganisms such that they can perform specific tasks autonomously or semiautonomously in complex environments [7]. The concept of using miniaturized devices or machines in medical applications was first visualized in 1966 in the popular science fiction movie Fantastic Voyage [6,8]. In this movie, a submarine was shrunk to a microscopic size and injected into the bloodstream of a person to remove a clot in their brain.

Particularly, in the medical field, microrobots could be used to perform specific tasks, such as drug delivery [9,10,11], targeted virotherapy [12], cargo delivery [13], and diagnosis and treatment [14]. The development of microrobots or micromachines that can replicate the sophisticated behaviors of microorganisms is a long-sought goal in the micro/nanorobotics field [7]. To enhance the therapeutic effect disease-specific drugs, it is important to achieve precise drug delivery. A microrobot that can be used as a drug-delivery vehicle must possess several capabilities, including a propelling force, controlled navigation, cargo-towing and release, and tissue penetration [15]. Apart from medical applications, microrobots can be used in several other domains, including water treatment [16], environmental remediation [17], and sensing and actuation [18]. The development of miniaturized microrobots by using photolithography, micromolding, and three-dimensional (3D) printing technologies, as well as chemical self-assembly approaches, has been ongoing for quite some time. Nevertheless, an intensive microrobot-application-specific investigation of material development and processing techniques is needed; these microrobots may be small in size, flexible, and capable of carrying out one or more tasks, but there are occasions when the combination of these qualities is not feasible due to current technical constraints. Novel materials and manufacturing techniques are crucial for giving microrobots all the qualities they need to retain the intended function while shrinking in size [19].

By definition, 4D printing can be defined as a process that facilitates structural transformation over time [20]. In contrast to 3D printing, four-dimensional (4D) printing allows the base material to change its shape with time and under external stimuli with the aim of enhancing its functionality [21,22,23]. In addition, materials that are “smart” or “intelligent” are required to create 4D-printed objects [24]. Although there is not a single description of these materials, many scientists believe they can perceive stimuli and respond significantly. The transformation can affect its shape, functionality, or physical property [20,25]. Shape change is often observed in nature in response to external factors such as changes in the environment [26]. For instance, *Mimosa pudica* or the sensitive plant changes its shape in response to an external stimulus such as touch. There are two categories of shape morphing, namely 2D-to-3D and 3D-to-3D [27]. In 2D-to-3D shape morphing, a flat structure folds into a 3D shape. In particular, several stimuli-responsive polymers undergo physical or chemical changes in response to small changes in the environment. These materials can either self-assemble or undergo phase transitions or morphological changes [28] in response to several stimuli, such as temperature [29,30], pH [31,32], and solvents [18,33,34]. As a result of their shape-morphing abilities, these materials could be excellent candidates for use in multiple applications, such as micromachines, soft microrobots, artificial muscles, and cargo delivery [26].

With respect to 4D printing technology, remarkable results have been reported in various applications. For example, He et al. reported the use of 4D-printed starch-based purees made from sweet potato in food printing [35]. The authors reported spontaneous shape changes of 3D-printed purple sweet potato purees through microwave dehydration which removed the water from the printed structure and led to deformation. Another example, robust, versatile, and maintenance-free thermoelectric generators fabricated based on origami principles have been reported as solutions for energy harvesting at low temperatures [36]. A two-step origami folding procedure is used to create a printed thermoelectric generator by screen printing an n-type and a p-type thermoelectric material in a checkerboard pattern. Positioning them vertically creates a mechanically stable cuboid in which the heat flow happens in-plane with regard to the printed TE components.

**Figure 1:**
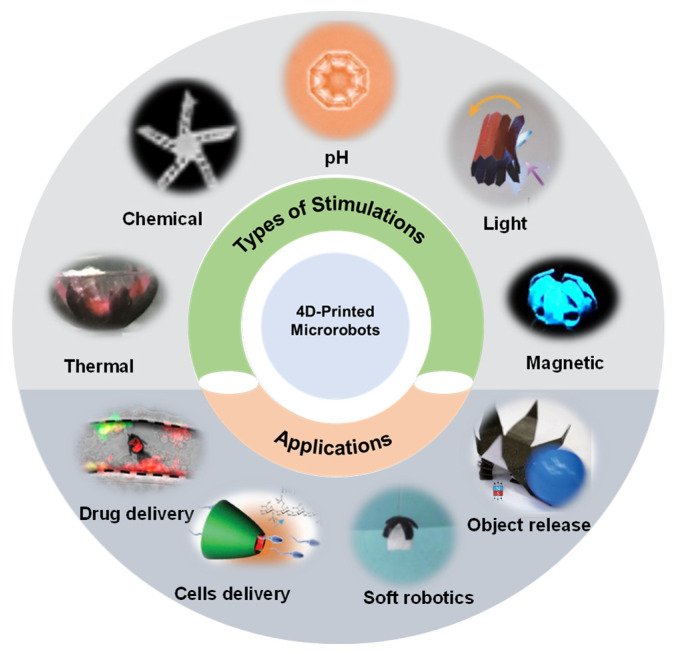
Overview of 4D-printed microrobots based on their stimulations and applications.

This self-aligning process makes insulation between the nearby thermocouples possible because of the substrate foil. Four-dimensional cardiac constructs sensitive to near-infrared (NIR) light have been reported for myocardial regeneration [37]. The temperature of shape memory polymers was elevated above its glass transition temperature, which softened the structure, decreased Young’s modulus, and deformed the materials. Zhang et al. reported the use of 4D printing technology in medical applications, including for the cell culture of HeLa cells and drug evaluation, by using the pH-responsive hydrogel [38]. Therefore, by simply altering the submerged environment between acidic and alkaline solutions, the sizes of microarchitectures could be reversibly transformed. In addition, 4D-printed microrobots have a broad size range, from large macroscale objects to micro/nanoscale devices [30,39]. As illustrated in Figure 1, researchers have studied various approaches to develop stimuli-responsive microrobots; those stimulations include thermal, chemical, pH, light and magnetic fields. Moreover, throughout the application of those stimulation, the microrobots could be developed for use in medical applications, such as for drug delivery, cells delivery, etc. [40,41,42,43], and for use in nonmedical applications such as for soft robotics, object carriers, etc. [18,44,45]. Additionally, multiple methods have been proposed to 4D-print microrobots by means of ultraviolet (UV) lithography, 3D printing, direct laser writing (DLW), and laser cutting, as summarized in Table 1. Overall, the research on microrobots is nascent. However, the domain is evolving rapidly and has the potential to revolutionize many areas—for instance, the medical and technology fields—in the coming years.

The main objective of this review article is to study the promising 4D-printed microrobots used in various applications. A brief introduction to microrobotics technology in the form of an overview of 4D-printed microrobot is in Section 1. Section 2 addresses the different materials used to fabricate microrobots. Section 3 introduces the stimuli required to realize various shape-morphing mechanisms. Section 4 describes the various possible applications of microrobots, while Section 5 describes the expected challenges in this field and future perspectives of 4D-printed microrobots.

## 2. Reversible Shape Transformation Strategies for Microrobots

There are several strategies for 4D-printing microrobots, including changing the number of layers used in the microrobot structure and selection of smart materials. Various materials such as plastic, metal, and ceramic have been used extensively to build 3D structures. However, these materials cannot be used in 4D printing because they do not react to stimulation [60]. Appropriate materials should be selected to build 4D structures, such as smart materials [61]. Because gels and hydrogels undergo large deformations owing to their high degree of swelling, they are especially attractive as active materials for building morphable structures [62]. In particular, the choice of photoinitiator plays an important role in the 4D printing system [63]. Therefore, it is important to choose a suitable photoinitiator for a given material. For instance, the photoinitiator must be soluble in water or polar solvents; must exhibit strong features in the two-photon cross section; and should have low toxicity, especially if it is to be used applied in biological applications [63]. The development of microrobots is mainly inspired by nature. Taking bacteria as an example, we can learn about bacterial propulsion, intelligence, programmability, and adaptability to environments [7,64]. Candidate materials for microrobot fabrication must fulfill several requirements because their properties will affect the application of the fabricated microrobots. For instance, biocompatibility is an important requirement in the fabrication of microrobots meant for biomedical applications [6].

The preliminary development and manufacturing of 4D soft robots that need to be propelled at such submicroscales begin with material selection [20]. Polymers have been used in microrobot fabrication because they are versatile in terms of functionality, softness, and recoverable strain, in addition to being lightweight [19]. Materials composed of smart soft polymers and gels react to external stimuli [65]. Among such polymers, hydrogels are the most popular. Hydrogels are multidimensional water-saturated polymer networks, and their water content can be as high as 99 wt.% [66]. The swelling behavior of hydrogels, which changes their size and volume, is their response to external stimuli, such as pH, ionic strength, temperature, and light [67]. In addition, the intrinsic properties of hydrogels, such as crosslinking density, microstructural anisotropy, and hydrophilicity, depend on their degree of swelling [68]. In this chapter, two types of microrobot fabrication strategies based on the number of layers are described.

### 2.1. Single-Layer Material

One of the main advantages of single-layer materials is their homogeneity, which means that they lack the irregularities that are mostly found in multilayer materials because of differences in the properties of the layer materials [69]. Additionally, by using single-layer materials, the preparation and printing times can be shortened. Furthermore, one of the most important properties of microrobots is their actuation system, and several researchers have reported that the selected microrobot material should be mixed with magnetic nanoparticles in a single layer to facilitate controlled shape transformation of microrobots under a magnetic field [33,58]. In [18], a single-layer microrobot was fabricated and studied. This microrobot was fabricated using UV light, and various microrobot shapes were patterned. Moreover, reversible shape transformation of the microrobot was demonstrated for folding and unfolding in ethanol/methanol and dimethyl sulfoxide (DMSO)/ethyl acetate, respectively. Zhang et al. demonstrated the fabrication of stimuli-responsive microarchitectures by using acrylic acid (AAc) as the monomer, pentaerythritol triacrylate (PETA) as the cross-linker, and 4,4′-bis(diethylamino) benzophenone (EMK) as the photoinitiator [38]. By creating homogeneous hydrogel photoresists, they were able to fabricate cubic, octagonal, and microclaw structures with shapes that could be tuned reversibly by simply changing the environment from acidic to alkaline and vice versa. 

### 2.2. Multilayer Material

The use of multilayer materials is another strategy for fabricating 4D-printed microrobots. In general, compared to single-layer materials, multilayer materials can provide more functionality or possess the ability to react to multiple stimuli. Many studies have been conducted to enhance the structural design of microrobots or ensure that they can provide multiple responses to diverse stimuli. By creating a structure composed of active and passive layers of SU-8 material, Su et al. demonstrated reversible shape morphing upon exposure to stimulation [47]. When the structure was stimulated with acetone and water, it folded and unfolded to its original shape, respectively. Rivera-Tarazona et al. demonstrated the 4D printing of an engineered living material (ELM, hydrogel) based on yeast-containing bio-inks and cell-free inks [70]. The top and bottom layers of this bilayer hydrogel were composed of the genetically engineered Saccharomyces boulardii mutant TRP-1 and URA3, respectively. The morphing ability of this bilayer hydrogel was attributed to the presence of a synthetic medium with either the amino acid L-tryptophan or nucleotide acid. In addition, bilayer hydrogels composed of individual layer that possess different properties or geometries exhibit asymmetric response behaviors and could be promising candidate materials in the pursuit of fast, sensitive, and tunable shape morphing [71]. Hao et al. used photolithography to fabricate a double-layer microrobot [31]. The first layer was composed of poly(ethylene glycol) diacrylate (PEGDA) with iron (II, III) oxide particles (Fe_3_O_4_) to facilitate magnetic locomotion, and the second pH-responsive layer was composed of 2-hydroxyethyl methacrylate (PHEMA). This bilayer hydrogel could be deformed by inducing different volume changes in the first and second layers [72]. Baker et al. 3D-printed trilayer structures composed of a polyurethane hydrogel core and polyurethane elastomer skins [73]. Discrete localized gaps in the elastomeric skins were designed to function as active hinges. Several complex origami fold patterns were produced depending on the spatial distribution of these hinges as a consequence of shape changes induced by hydration.

## 3. Stimulations

Several external stimuli, such as pH, ionic strength, light, heat, characteristics of the swelling agent, and electric and magnetic fields, could be used to trigger the shape morphing of microrobots [26]. In this section, we describe these stimuli.

### 3.1. Thermal

**Figure 2:**
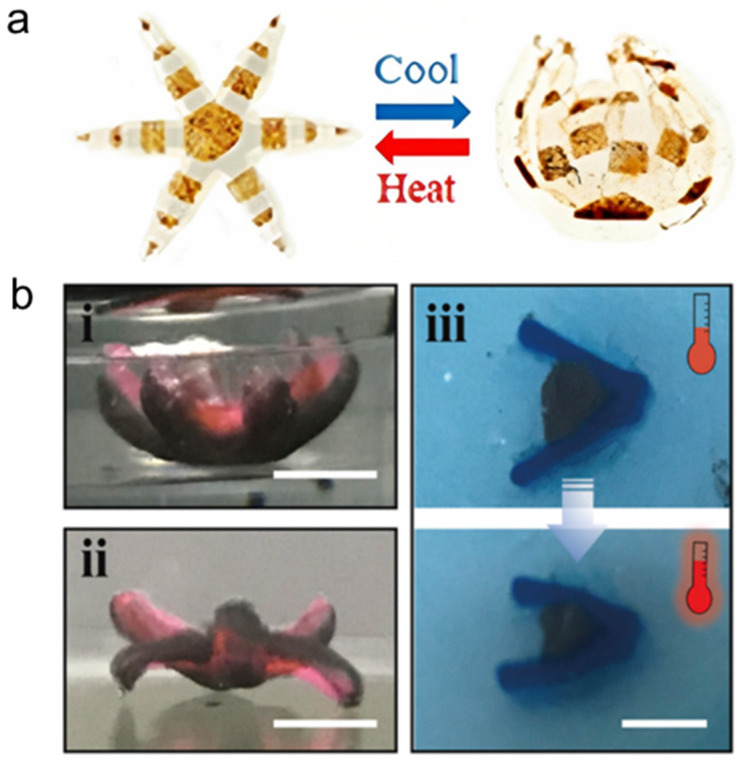
Temperature-responsive microrobot: (**a**) Shape change of a gripper in response to changing of temperature. Reprinted with permission from Ref. [46]. Copyright 2019, American Chemical Society. (**b**) Shape transformations of various printed structural configurations. (i) A leptasteria-like gripper with six identical deformable directional lappets. (ii) A dual-head gripper with two opposite deformable directional lappets. (iii) A gripper simulating shellfish with different cross-linking densities in the vertical direction. Reprinted with permission from Ref. [75]. Copyright 2022, Elsevier.

Temperature is one of the most widely employed stimuli for shape-shifting in polymer-based materials [74]. A thermoresponsive microrobot was developed using poly(oligoethylene glycol methyl ether methacrylate-bis(2-methacryloyl)oxyethyl disulfide) (Mn = 500) [P(OEGMA-DSDMA)] and a relatively nonswellable poly(acrylamide-N, N′-bis(acyloyl)cystamine), P(Aam-BAC) passive gel [46]. This microrobot was biodegradable, and its shape transformation occurred between 50 and 70 °C, as illustrated in Figure 2a. This shape transformation was attributed to the P(OEGMA-DSDMA) gel becoming hydrophobic above its volume transition temperature, which caused the microrobot to unfold; by contrast, below the volume transition temperature of the gel, the microrobot folded. In addition, many researchers have studied thermal stimulation. Hu et al. fabricated a bilayer of small robots that deformed in response to changes in temperature [75]. The first layer consisted of a thermosensitive hydrogel (TSH) based on N-isopropylacrylamide (NIPAM) monomer, and the second layer consisted of NdFeB magnetic particles embedded into the TSH (MTSH). A dual-head gripper with two opposed deformable directional lappets and a gripper resembling a leptasterias with six similar deformable directional lappets were fabricated using this material (Figure 2b). The presence of magnetic particles in the structure hindered the flow of water in and out of the material, which had a marginal effect of the crosslinking density of the material. When pNIPAM exceeded the low critical solution temperature (LCST), the bonding between the hydrogel and water was cleaved. Consequently, the hydrogel shrank and the shrunken volume of TSH was 3.73% higher than that of MTSH.

Lee et al. developed microdevices that were fabricated from polyNIPAM (pNIPAM) hydrogel by using photolithography [76]. The shape-morphing ability of this material was attributed to the dehydration-induced stress between the soft and hard layers. The folding angle of this material was proportional to the density difference between the soft and hard layers. Additionally, in thermally stimulated bilayer materials, the shape change effect occurs when the difference between the thermal expansion coefficients of the constituent layers is adequately large [77]. Nojoomi et al. also developed a temperature-responsive microrobot [78]. They proposed a two-dimensional (2D) material programming approach to fabricate shape-morphing 3D structures, including stingray structures based on thermoresponsive materials, and demonstrated the swimming motion of these structures in two different modes. Metamaterials were used to fabricate thermoresponsive microrobots [79]. Under electron beam heating for 10 s, the fabricated structures exhibited a thermomechanical response in the form of motion. The device was well controlled by a straightforward thermomechanical process (thermal expansion) in a dry environment, and it provided precise motion amplitudes and reversible responses. Although the temperature is versatile, meaning it may be used in both medical and nonmedical fields, raising the temperature takes time, which results in a slightly slow response and could limit the application of this type of microrobot. 

### 3.2. Chemical

**Figure 3:**
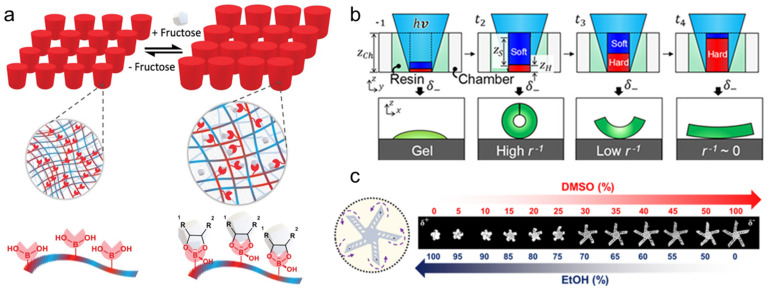
Chemical-responsive microrobot: (**a**) Illustration depicting the mechanism of hydrogel expansion. Reprinted with permission from Ref. [48]. Copyright 2023, Wiley-VCH. (**b**) Schematic diagram depicting optimization of the proposed system. (**c**) Showcase experiments conducted to test the reversibility of shape morphing under stimulation. Reprinted with permission from Ref. [18]. Copyright 2021, American Chemical Society.

Several studies have demonstrated the use of chemical solutions to stimulate shape changes in microrobots. A novel sugar-responsive microrobot, illustrated in Figure 3a, was fabricated from a phenyl-boronic acid copolymer [48]. Owing to the creation of regions with different degrees of swelling, the fabricated structure could undergo reversible expansion and shrinkage in response to contact with fructose and phosphate-buffered saline (PBS) solutions. The fabricated structures responded rapidly to fructose because the immobilized phenylboronic acid (PBA) moieties along the hydrogel network yielded a boronate ester when exposed to sugars in an aqueous medium. This caused swelling of the network owing to the repulsion between the negatively charged boronate ester to fructose solutions. This indicated that the material became hydrophilic after binding to sugar.

In addition, Li et al. demonstrated a 4D-printed microrobot that can be stimulated by using a chemical solution [18]. By means of UV lithography, they fabricated a five-arm gripper microrobot and stimulated it using ethanol and DMSO. The results indicated that the robot’s shape could be morphed when the thickness ratio of the UV-cured soft and hard layers reached a certain value (Figure 3b). In addition, the density of the bottom layer was typically higher than that of the top layer because UV radiation was concentrated on the top layer, resulting in higher light intensity. As a consequence, the 2D UV-printed membrane folded into a 3D form (Figure 3c). Similarly, Su et al. fabricated a soft active polymer by introducing a swellable guest medium into a nonswellable host polymer matrix [47]. The authors demonstrated a fabrication method that created passive and active regions in the 3D-printed SU-8 material. Patterned samples fabricated from this material folded when stimulated with acetone and unfolded to their original shape when stimulated with water. This was attributed to the different thermal expansion coefficients of the passive and active layers, and while these expansion coefficients determined the bending direction, they had a limited effect on the bending curvature. Su et al. printed two different geometries. Their findings indicate that as the pattern thickness increased, the response time virtually doubled. When the pattern thickness was 330 mm, the time required for the material to curve was 95 s, but when the pattern thickness was extended to 607 mm, the curving time increased to 232 s. Additionally, the 4D-printed microrobot that can be triggered by chemical solution responds quickly to stimuli, although the usage of chemical solution may constrain its usage in medical fields. 

### 3.3. pH

pH-responsive microrobots can swell or shrink at certain pH values owing to the protonation of ionizable groups or degradation of acid cleavage bonds, which cause the polymer chains to degrade [80,81]. pH-responsive material has been extensively studied due to its excellent biocompatibility, adjustable toughness, high water content, and reversible volume change [82]. Acrylic acid (AAc) is typically used as the copolymer to initiate pH-responsiveness [83]. The AAc incorporated in the responsive layer increases the swelling as the pH of the medium increases. This is because in a basic environment, carboxylic acid groups (–COOH) deprotonate to form negatively charged carboxylate ions (COO–), which repel one another and absorb water to fill the porous network [84]. In another study, a separate bilayer microrobot that can be stimulated by pH was reported [85]. The authors of this study demonstrated the behavior of this 4D-printed microrobot under pH values of 6 (folding) and 11 (unfolding). The strain mismatch between the top and bottom layers, resulting from the difference in the extents of swelling between the hydrogel with a high degree of crosslinking and that with a low degree of crosslinking stripes, caused the unwinding deformation. Similarly, Darmawan et al. fabricated a pH-responsive microrobot for targeted drug delivery [50]. This microrobot employed a commercial photoresist and pHEMA/AAc hydrogel. Hydrogels that are pH-stimuli contain ionizable functional groups that can receive or release protons in response to changes in the pH of the surrounding environment. It exhibited the ability to fold and unfold at certain pH values. The authors exposed the microrobot to pH > 7 to initiate folding; thereafter, it was exposed to a lower pH (pH < 7) to initiate unfolding while releasing the drugs to be delivered. Therefore, the proposed microrobot could be used for drug delivery, especially in stomach cancer therapy.

**Figure 4:**
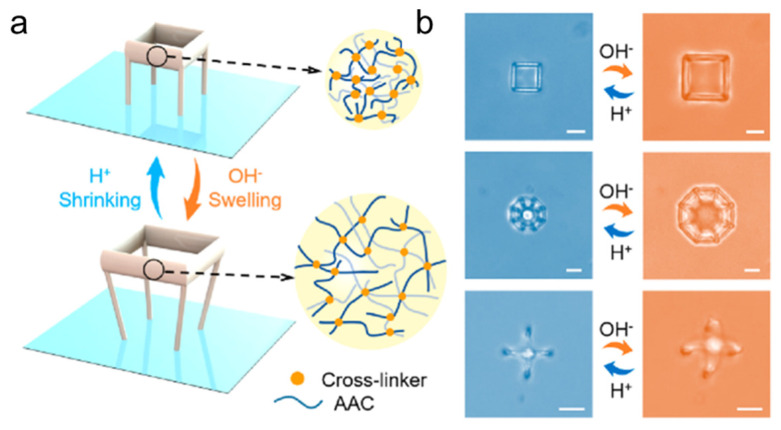
pH-responsive microrobots: (**a**) schematic diagram illustrating pH-responsive swelling and shrinking behaviors of various hydrogel architectures; (**b**) optical images depicting shape transformations of different hydrogel architectures: cubic (top), octagonal (middle), and inverted microclaw (bottom). Reprinted with permission from Ref. [38]. Copyright 2022, American Chemical Society.

Furthermore, Xin et al. fabricated pH-responsive microrobots of several shapes, including microfish, microcrab, and microbutterfly [49]. Due to internal stress brought on by variations in point densities, the fabricated structures swell in solution at a high pH and even exhibit modest reverse bending. In contrast, the structures morph at a low pH because the carboxylic groups of AAc release protons. In addition, because the scanning density was encoded in localized sections of a single type of pH-responsive hydrogel, the fabricated microrobots exhibited strong deformability. Another study on pH-responsive microrobots was conducted by Zhang et al. [38], in which they demonstrated a microstructure that exhibited reversible shape morphing when its aqueous environment was switched between acidic and alkaline (Figure 4a). Similarly with the abovementioned, shape changes brought on by the ionization and deionization of carboxylic groups in AAc when exposed to environmental pH variations. In addition, they demonstrated the shape reversibility of several other structures, as illustrated in Figure 4b, that were prepared using the dynamic holographically shifted multifoci method. These structures swelled and unswelled within 300 ms when exposed to different environmental pH, which indicated that rapid diffusion of acid or base occurred in the structures. Wang et al. reported an inverse design approach for building micromachines capable of achieving tailored shape deformations in two-photon polymerization processes [86]. pH-responsive hydrogels and photopolymeric resins, used as active and inactive materials in heterostructure construction, respectively, can sense pH changes in the surrounding media and cause bending deformations.

### 3.4. Light

**Figure 5:**
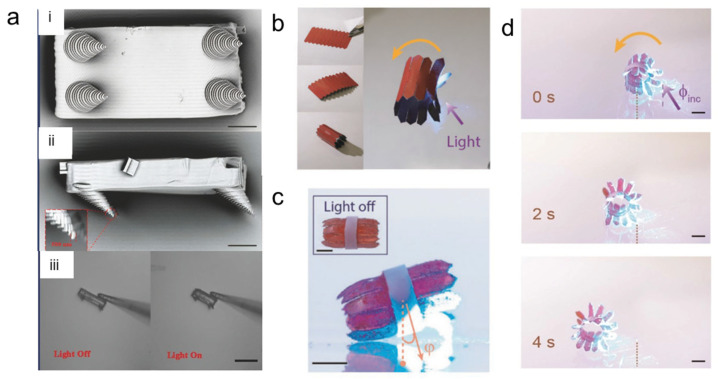
Light-responsive microrobots: (**a**) (i) Scanning electron microscopy image of microwalker lying upside down. Scale bar is 10 μm. (ii) Side view of the microwalker. Scale bar is 10 μm. (iii) Actuation of microwalker under laser beam radiation with 532 nm. Scale bar is 50 μm. Reprinted with permission from Ref. [55]. Copyright 2015, Wiley-VCH. (**b**) Design of light-propelled rolling robot. (**c**) Photograph of robot illuminated on one side of the body to induce tilt (angle φ). The inset shows the same robot when light is off. (**d**) Images showing multigait rolling motion along a straight line under irradiation through the bottom substrate from the direction of the violet arrow. Reprinted with permission from Ref. [53]. Copyright 2019, Wiley-VCH.

Another stimulus for altering the shapes of 4D-printed microrobots is light. Light irradiation can initiate chemical reactions that would otherwise not occur under ambient conditions, thereby acting as a remote control over space and time. Consequently, light is being utilized as a powerful tool to build polymeric materials and open up new avenues for technological advancement in various industries [87]. Shape transformation occurs because of material responsiveness to a certain range of UV or visible light wavelengths [88]. Zeng et al. demonstrated a light-stimulated walker microrobot based on artificial muscles composed of liquid crystalline elastomers [55]. To reduce the surface contact area, they employed a conical shape, and to achieve the adhesion asymmetry necessary for walking, the legs were tilted to 45°. Scanning electron microscopy images of the fabricated walker are shown in Figure 5a. The fabricated walker was able to undergo reversible deformation when exposed to a modulated laser beam by absorbing light from the overall environment.

Cheng et al. demonstrated a microrobot that can be actuated and changes its form when exposed to light [53]. The aforementioned robot consisted of light-responsive petals and was fabricated by following Kirigami principles (Figure 5b). On both sides of the central axis of a liquid crystal network (LCN) film, patterns composed of triangular-ended series of stripes were created. These stripes were laser-cut along the planar direction of the splay-aligned film, which ensured that they were flexible enough to bend under the influence of light and generate the locomotion’s photomechanical petals. Upon exposure to light, the petals tilt at an angle that depends on the amount of radiation applied, thus bending toward the substrate and raising the center of mass of the structure (Figure 5c). Meanwhile, all petals remain inactive and the body remains parallel to the ground when the robot is placed on a flat substrate in the dark (see the inset of Figure 5c). As can be seen in Figure 5d, oblique irradiation causes the actuating petals to roll into darkness, cool down, and unbend after the bottom petals deform and propel the robot forward. A fresh set of petals is exposed to light in the meantime, and they bend against the ground to propel the robot forward. In addition, the photomechanical petals can function as a control movement for moving backward or forward, and can climb up to 6°. Another example of a light-responsive microrobot is reported by Deng et al. [54]. The crosslinked network of the composite hydrogel’s mechanical modulus is simultaneously increased by the homogeneous doping of single-walled carbon nanotubes (SWNTs) inside NIPAM, increasing its thermal conductivity, light absorption coefficient, and mechanical modulus. This solves the problem of the well-known material trade-off between the mechanical modulus and the response sensitivity of stimuli-responsive hydrogels. Due to its excellent intrinsic qualities, the light-responsiveness has a variety of special benefits, including the ability to be precisely and remotely driven and the ability to modulate a multidimensional light field such as wavelength, frequency, intensity, polarization state, and spatiotemporal distribution of energy [54]. However, the light-induced actuation capabilities of microstructures are severely constrained by the low light absorption coefficient and poor heat conductivity of the common stimuli-responsive hydrogel materials.

### 3.5. Magnetic

**Figure 6:**
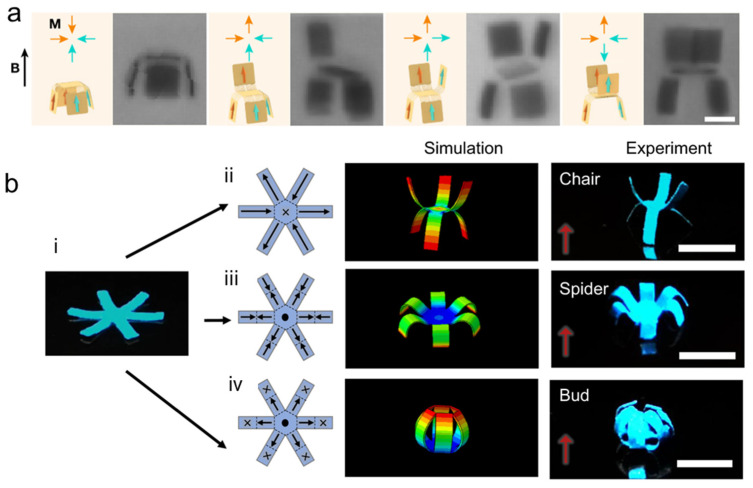
Magnetic-responsive microrobots: (**a**) Schematics of the magnetic configurations and shape morphing behaviors of the micromachines upon application of the controlling magnetic field B = 15 mT; the optical microscopy images depict the four different conformations of the fabricated devices. Scale bar is 10 μm. Reprinted with permission from Ref. [89]. Copyright 2019, Springer Nature. (**b**) (i) six-armed magnetic shape-morphing material film. Schematic illustrations of magnetization pattern, finite-element simulations, and optical images of six-armed magnetic shape-morphing material depending on magnetization patterns can be transformed to (ii) Chair. (iii) Spider. (iv) Bud. Scale bar is 5 mm. Reprinted with permission from Ref. [92]. Copyright 2020, Springer Nature.

Robotic systems that are stimulated by magnetic fields have been reported in the literature. For instance, under magnetic fields of 200 mT, several 3D-structure-based devices exhibited rapid and reversible transformation [58]. The application of a magnetic field induced torques in the embedded ferromagnetic particles, and the stresses generated as a result induced a response. Under magnetic fields of 200 mT, the authors were able to produce a series of high-aspect-ratio multilayered structures that underwent rapid, reversible transformations between complicated 3D geometries owing to the use of support ink and the resulting capacity to print 3D structures with programmed domains. The craft of paper folding, origami, served as inspiration for the design of these micromachines, which featured two different types of parts: stiff panels, some of which were functionalized with arrays of single-domain nanomagnets covering the panel surface, and structured “soft” spring hinges serving as the connecting creases [89]. Figure 6a shows a schematic illustration and optical microscopy images of the shape-morphing behavior of these micromachines under a magnetic field of 15 mT. In another study inspired by origami, a soft magnetoactive robot that consisted of magnetizable neodymium-iron-boron particles embedded in a polymer resin was developed [90]. The fabricated machines that have been fully magnetized retain their residual magnetic polarity patterns, i.e., magnetization (M). Thus, the machines have actuation magnetic fields that cause them to move and deform. In addition, the authors of this study conducted a locomotion test in which this robot was able to perform the tasks of deployment, locking, sequential folding, and cargo transportation.

Another robot featuring programmable shape change was developed by Xu et al. [91]. This magnetic-field-responsive robot was composed of a magnetic composite material, and it was fabricated using the UV lithography method. It exhibited large angle bending; combined bending and torsion; and higher-order, multiaxis deformation. Alapan et al. developed a magnetic microrobot composed of chromium dioxide (CrO2) microparticles (average diameter = 10 μm) embedded in a polydimethylsiloxane (PDMS) elastomer [56]. Under heat-assisted magnetization, this microrobot was able to undergo high-resolution shape transformation. The deformation of connected segments due to complementary 3D magnetization can lead to the development of closed structures. Under a 60 mT magnetic field, planar segments joined by hinges were magnetized in both the in-plane and out-of-plane directions to form a closed cube. In Figure 6b, Deng et al. demonstrated an origami magnetization pattern with different angles in six-armed magnetic-responsive soft material [92]. Inspired by Pangolin, Soon et al. developed an untethered magnetic small robot that could perform shape morphing [57]. Here, an overlapping scaled design enables users to simultaneously realize on-demand thermal functionality in combination with shape-morphing and mobility capabilities. Its untethered control, quick reaction, and extensive penetration range are the advantages of a magnetic field [93]. However, using permanent magnets in the structure restricts the robot’s flexibility and ability to be more compact, while raising the unfavorable chance of magnet failure in medical applications [94].

### 3.6. Hybrid

Several studies have demonstrated microrobots that can be actuated by multiple stimuli. For instance, Lee et al. reported a microrobot that was fabricated using a 3D printer and was responsive to temperature, pH, and divalent cations over multiple cycles [95]. The authors formulated a precursor mixture of pNIPAM and acrylic acid in the form of a copolymer to initiate those stimuli. Below its LCST, the polymer chains of pNIPAM were hydrophilic and established hydrogen bonds with water molecules, which expanded the structure. Additionally, because of the transition between its protonated and deprotonated states and charge screening, the carboxylic acid groups on the acrylic acid were able to respond to pH and the presence of divalent ions, respectively. In another study, Yang et al. developed a multiresponsive fish-like microrobot composed of pNIPAM, carbon nanotubes (CNTs), and magnetic nanoparticles (MNPs) [59]. This robot was able to rise and sink, travel along a specified track, and swim fast on the surface of water when exposed to a xenon light, magnetic field, or ethanol molecules to execute diverse tasks. Both the crosslinked pNIPAM hydrogel and its aqueous solution exhibited temperature-sensitive characteristics because of the hydrophilic amide group and hydrophobic isopropyl group in the macromolecular chain of pNIPAM. Additionally, the CNTs on the surface of the microrobot absorbed light energy from the xenon light and transformed it into heat, which altered the surface tension of the surrounding ethanol. This generated a buoyant flow that drove the movements of the aforementioned microrobot.

## 4. Applications

Thus far, we have described how 4D-printed microrobots have promising applications as smart microrobots owing to their shape-morphing abilities and the diverse types of constituent materials and stimuli that can be used. Therefore, such microrobots could be used in medical and nonmedical applications.

### 4.1. Medical Applications

**Figure 7:**
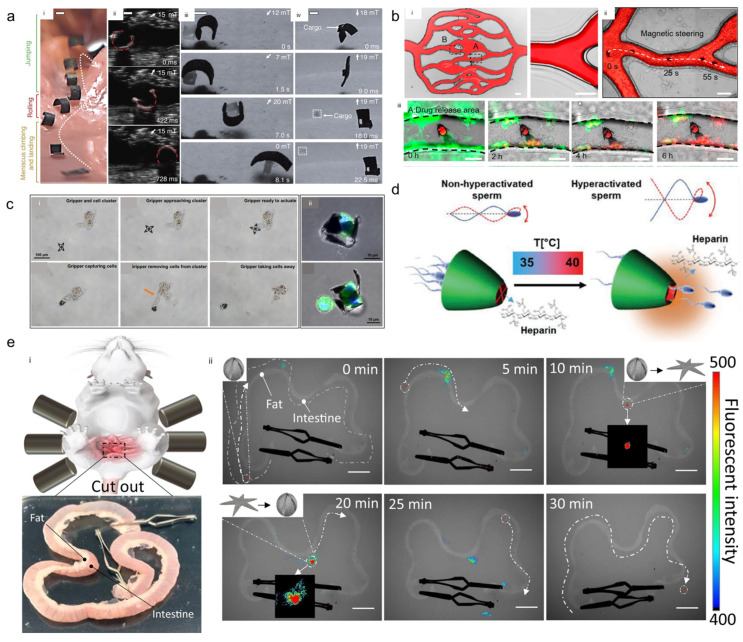
Medical applications of 4D-printed microrobots: (**a**) (i) Soft robot navigating across a synthetic stomach phantom by using a combination of locomotion modes. (ii) Ultrasound-guided locomotion whereas the robot rolls within the chicken tissue and visualized by ultrasound. (iii) Robot approaching a cargo item by walking on a flat rigid surface, picking up the cargo by curling into the C-shape, transporting the cargo away and maintaining the shape, and releasing the cargo at targeted area. (iv) Dynamic and selective cargo release. Scale bars are 1 mm. Reprinted with permission from Ref. [100]. Copyright 2018, Springer Nature. (**b**) (i) Artificial networks fabricated by photolithography and PDMS molding. (ii) Magnetic shape-morphing microfish swimming in these channels. (iii) Snapshots of the HeLa cells viability in the doxorubicin-release area. Reprinted with permission from Ref. [49]. Copyright 2021, American Chemical Society. (**c**) (i) Demonstration of cell biopsy from a cell cluster by gripping the cell. (ii) Immunofluorescence images of cells captured by the grippers. Reprinted with permission from Ref. [102]. Copyright 2015, American Chemical Society. (**d**) Schematic illustration of heparin release and sperm hyperactivation. Reprinted with permission from Ref. [103]. Copyright 2022, Wiley-VCH. (**e**) (i) Ex vivo model of a rat intestine. (ii) Demonstration of ex vivo transformation and transportation of ionic shape-morphing microrobotic end-effectors carrying MNPs and fluorescent microbeads through external magnetic field control. Scale bar is 2 cm. Reprinted with permission from Ref. [104]. Copyright 2021, Springer Nature.

Additionally, owing to their small sizes, microrobots have great potential for use in various medical approaches [5]. Many researchers have developed medical microrobotic applications (Figure 7). In addition, biocompatibility and biodegradability plays important roles in designing the medical microrobot [96,97]. In contrast to passive nanoparticle drug carriers, microrobots can penetrate thick healthy, and malignant tissues. Therefore, the applications of 4D-printed microrobots in medical fields will be discussed as follows. 

#### 4.1.1. Biopsy

Because of their distinctive dynamic properties, stimuli-responsive materials can either supplement or entirely replace real tissues. For this reason, stimuli-responsive materials have been developed since a long time to fulfill the requirements of biomedical devices [98,99]. Hu et al. demonstrated a soft magnetoelastic small robot that can roll and walk on solid surfaces, climb liquid menisci, swim inside and on the surface of liquids, jump over obstacles, and crawl into small spaces [100]. They demonstrated the locomotion of a microrobot in a surgical human stomach phantom, and this soft robot was able to grab, carry, and release an object into the targeted area, as shown in Figure 7a. 

#### 4.1.2. Drug Delivery

Microrobots can incorporate sensing tools that can identify elements of the chemical environments of tumors and assist in the accumulation of said sensing tools in tumors [101]. One of the promising applications of microrobots is targeted drug delivery. Such microrobots carry specific drugs or cells through the body and release them in targeted areas [31,50,75]. By using microrobots for drug delivery, the side effects of conventional therapy could be mitigated while improving the effectiveness of drugs, as has been demonstrated in several studies by using 4D-printed microrobots. Additionally, Xin et al. demonstrated drug encapsulation and controlled drug release from pH-responsive microfish [49]. This microfish has a stable mouth that morphed at cell culture temperature (37 °C), and it entrapped drugs in its mouth when the environment pH was less than 7. Finally, when the entrapped drugs were released from the microfish (pH < 7), they were able to kill cancer cells (Figure 7b).

#### 4.1.3. Cells Delivery

Breger et al. fabricated double-layered gripper-shaped microrobots that could be stimulated thermally and controlled by applying a magnetic field [42]. The grippers were stored at 4 °C such that they folded entirely because of maximum water absorption by the pNIPAM-AAc layer. Pipetted on top of a fibroblast cluster, the grippers were allowed to open and close in warm PBS (37 °C) around the tissue, and those grippers were able to grip it. For visualization purposes, the grippers are marked with a dotted white line. The grippers were retrieved using a pipette and examined microscopically. The authors identified cells within the grippers’ arms after retrieval. In Figure 7c, Jin et al. demonstrated the active biopsy of cells through gripping the cells by using thermoresponsive grippers [102]. Initially, the gripper was moved magnetically to approach the cells cluster. Once the grippers positioned properly, the temperature was increased to induce the gripper to close and capture cells. The process highlights the agility of motion and firm grasping, which are essential for surgical operations. 

A 4D-printed sperm-hybrid microcarrier fabricated from a thermoresponsive pNIPAM hydrogel was evaluated for use in assisted reproduction [103]. The authors reported that this 4D-printed sperm microcarrier employed both a non-stimuli-responsive polymer (IPS photoresist) and a thermoresponsive hydrogel (pNIPAM). They demonstrated the capture, transport, and release of sperm cells in the targeted area by applying an electromagnetic field to control the motions of the microcarriers. The temperature was fixed to 35 °C during the entire transportation process. Once the microcarrier reached the last reservoir, the temperature was increased to 40 °C. Owing to this increase in temperature, the microgate of the microcarrier opened, which allowed the sperm cells to be released one at a time (Figure 7d). Zheng et al. developed a 4D-printed microrobot for ex vivo manipulation of rat intestine, as shown in Figure 7e. Initially the microrobot was in a folded state and located on the left side [104]. As the magnetic field was applied, it moved along the middle part of the intestine. Upon the application of a stimulus, it performed self-release (unfolding) within 10 min, and when CaCl_2_ was injected, it transformed back to its initial state and moved farther from the intestine. This demonstrated that the aforementioned microrobot can be used in in vivo applications.

#### 4.1.4. Drug Treatment Evaluation

In another example, in terms of drug treatment evaluation, a pH-responsive microrobot was used to observe the efficacy of a drug used to treat HeLa cells [38]. By tracking the fluorescence of the cells in an array, it was possible to determine the therapeutic effects of various Doxorubicin concentrations. Drug efficacy was determined accurately by comparing the observed variations in cell-inhibitory effects between microrobots loaded with various drug concentrations. A further indication of the possibility for long-term cancer inhibition in practical treatment is the persistent release of medicines from the microrobot, which can limit cell development in sizeable regions outside the structural array.

### 4.2. Nonmedical Applications

**Figure 8:**
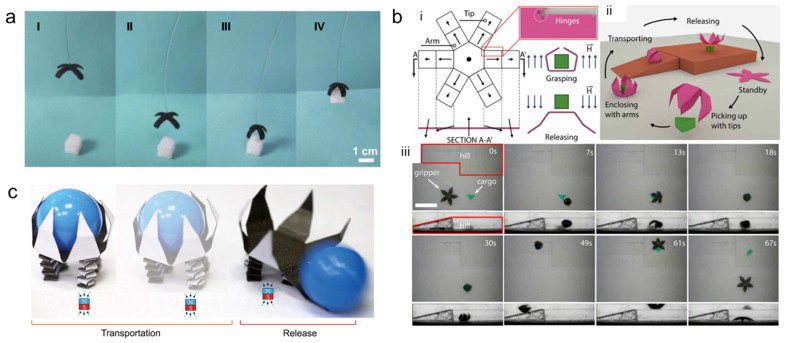
Nonmedical applications of 4D-printed microrobots: (**a**) (I–IV) After being exposed to the NIR light for two seconds, the gripper shrank and captured the target. Reprinted with permission from Ref. [14]. Copyright 2022, IOP. (**b**) (i) Geometry, magnetization profile, and working mechanism of a magnetic microgripper. The black arrows indicate the direction of local magnetization of each part; meanwhile, the blue arrows indicate the actuating magnetic field. (ii) Illustration of gripper to perform cargo delivery. (iii) Top and side-view images of cargo delivery of gripper in silicon oil. Scale bar is 5 mm. Reprinted with permission from Ref. [91]. Copyright 2019, Science. (**c**) Dynamic locomotion and on-demand cargo release by magneto-origami quadruped robot. Reprinted with permission from Ref. [90]. Copyright 2022, Springer Nature.

Four-dimensional-printed microrobots have attracted significant attention from the perspective of their application in nonmedical fields as well, especially in the field of actuators, owing to their shape reversibility that can be triggered by external stimuli (Figure 8). Thus, the applications of 4D-printed microrobots will be discussed.

#### 4.2.1. Soft Robotics

The capability to grasp an object is one of the advantages of responsive and untethered microrobots [91]. Zhan et al. developed a light-responsive hydrogel based on the photothermal effect and processed it using a projection microstereolithography (PSL)-based 3D-printing process to fabricate a microrobot with high reactivity and a simple control approach [14]. The shape and swelling motion of this hydrogel were controlled easily by varying the light intensity and irradiation position. Bending deformation of the crossing beams under NIR irradiation on the surface of the gripper was attributed to the variable swelling ratios of the asymmetric structure. After irradiation with NIR light, the gripper shrank in 2 s and grasped the object, which demonstrated a potential application of the printed gripper as a microrobot in object grasping and transportation (Figure 8a). 

#### 4.2.2. Cargo Delivery

A 4D-printed microrobot that was responsive to light and actuated by a magnetic field was developed and reported by Li et al. [105]. This microrobot was fabricated by embedding nickel nanowires in a photoactive hydrogel. The authors demonstrated walking, steering, climbing, and cargo delivery to any arbitrary destination under the influences of an external magnetic field and light. As shown in Figure 8b, Xu et al. fabricated a magnetically actuated six-degrees-of-freedom microgripper by UV photolithography [91]. To encapsulate the cargo of interest, the microgripper is equipped with a base section and numerous arms. When the arms are folded, the microgripper can be described as a rigid object that moves by rolling or when it is pulled by a magnetic gradient. The microgripper applies pinching forces at it tips, flips itself over, and firmly grasps the cargo. The authors demonstrated a rolling of the gripper to the target location, changed the magnetic field’s polarity to release the cargo, and then rolled it back to the starting position. As described above, 4D-printed microrobots show great potential for use not only in medical applications but in nonmedical applications. 

Li et al. demonstrated a microrobot that was able to grab and release an object when stimulated using a chemical solution [18]. The fabricated microrobot grabbed the object when the environmental solution was ethanol and released it when the solution was changed to DMSO. Similarly, Yi et al. developed a magneto-origami quadruped robot by combining a deployable flower gripper and a spring actuator, as illustrated in Figure 8c [90]. The robot’s legs, which allowed the gripper to securely grab the payload, were fitted with four spring actuators. The two front legs move to facilitate conveyance of the grabbed goods. When the magnet is moved forward, the frontal legs bend first. Then, with slight movements of the magnet, the robot moves forward by dragging its bent frontal legs. This quadruped robot can stride with a displacement of 2 mm in less than 2 s in a gait circle. Moreover, it can move forward quickly by repeating the gait circle. To ensure that the cargo is released on schedule, the magnet is positioned to be closer to the flower gripper as it approaches the final location.

## 5. Future Challenges and Perspectives

Over the past few years, advanced microrobot technologies have been developed using diverse materials and fabrication techniques, which has led to increased use of microrobots in various applications. Many studies have highlighted the potential for using microrobots in the near future. In addition, 4D-printed microrobots prepared from responsive materials undergo structural or functional changes when triggered by various stimuli. In this work, we have provided an overview of 4D-printed microrobots by focusing on the strategies for manufacturing them, including varying the number of layers and typical stimuli for initiating shape-morphing behaviors. In addition, we have discussed the prospective applications of the aforementioned microrobots.

While 4D-printed microrobots hold promise for a range of applications, there is a need to enhance the efficiency of current fabrication techniques. Proper control of printing parameters, like thickness and orientation, is crucial to achieve desired four-dimensional printing behavior [30]. Moreover, response to stimulus is the key characteristic of 4D-printed microrobots. Thus, smart materials with an efficient fabrication process will allow us to achieve more versatility and enhance the ability of microrobots to respond to stimuli. Overall, multidisciplinary studies, including the use of smart materials and novel fabrication methods, are required to develop more advanced 4D-printed microrobots. Moreover, further studies on robotics performances, including robot movement accuracy and microrobot design, should be conducted to fulfill the requirements of various application fields. In addition, 4D-printed microrobots must be stimulated to actuate their shape-morphing behaviors. In this respect, it is important to control their stimulation to achieve faster and more versatile responses. Furthermore, combining modeling or simulation with optimization algorithms enables the identification of structural solutions that achieve the desired deformation in a broader trial-and-error space [106]. As an outcome, this method is emerging as a promising design strategy.

Despite the reviewed studies that have demonstrated various therapeutic applications of microrobots, clinical translation of said applications remains slow. Biological systems are dynamic, and they can change easily when the environment changes, which seems to the be reason underlying the slow uptake of microrobots in clinical settings [107]. As a matter of fact, the reported microrobots exhibit excellent biocompatibility, but additional analyses and observations of medical microrobots are required to overcome the current inefficiencies. Nevertheless, the introduction of magnetic nanoparticles to control the movements of microrobots and the addition of contrast agents could increase the use of microrobots in medical applications [108,109]. More importantly, biodegradability of the proposed microrobots is essential, and this aspect remains challenging [97]. In this light, the development of a 4D-printed microrobot by using biodegradable ink materials could be the solution to this problem.

The emerging 4D printing technology can be enhanced by combining it with machine learning and artificial intelligence. By applying these technologies for tasks ranging from sensing to decision making, the optimum settings for applying 4D-printed microrobots in real-world applications can be determined. In sum, the 4D printing of microrobots is an interdisciplinary endeavor that involves the use of multiple skillsets to develop and fabricate an on-demand dynamic microrobot structure. A foundation for 4D-printed microrobots to be used in both medical and nonmedical applications has been established by the astounding studies conducted in previous decades. The development of 4D-printed microrobots will usher in a new generation of smart microrobotic systems that can be used in the next generation of advanced micromachines.

## Figures and Tables

**Table 1:** Overview of 4D-printed microrobots.

Main Material	Fabrication	Layer	Geometry	Stimulation	Ref.
NIPAAM	UV	Multi	Gripper	Thermal	[42]
P(OEGMA- DSDMA)	UV	Multi	Gripper	Thermal	[46]
NIPAAM/PEGDA	UV	Multi	Helix	Thermal	[45]
SiO/SiO_2_	UV	Multi	Gripper	Thermal	[41]
NIPAAM/PEGDA	UV	Multi	Gripper	Thermal	[29]
E-dent	UV	Single	Gripper	Chemical	[18]
SU-8	3D printing and UV	Single	Gripper	Chemical	[47]
Acrylamide	DLW	Single	Vase	Chemical	[48]
E-dent	UV	Single	Gripper	Chemical	[34]
NIPAAM/AAc	DLW	Single	Microfish	pH	[49]
AAc	DLW	Single	Microcage	pH	[38]
E-dent/pHEMA	UV	Multi	Gripper	pH	[50]
NIPAAM/AAc	DLW	Single	Trumpet	pH	[51]
pHEMA/PEGDA	UV	Multi	Gripper	pH	[31]
pNIPAAM/AAc	3D printing	Multi	Pollen	pH	[52]
Liquid crystal polymer	Laser cutting	Single	Tube	Light	[53]
CNT/NIPAM	DLW	Single	Gripper	Light	[54]
Liquid crystal elastomers	DLW	Single	Cone	Light	[55]
CrO_2_/PDMS	Laser cutting	Single	Cube	Magnetic	[56]
Magnetic PDMS	Laser cutting	Multi	Rectangular	Magnetic	[57]
Silicon SE1700	3D printing	Single	Hexapedal	Magnetic	[58]
pNIPAM/CNT	UV	Single	Fish	Hybrid	[59]

## Data Availability

Not applicable.
